# Dermatomyositis revealing breast cancer: report of a case

**DOI:** 10.11604/pamj.2015.21.89.6971

**Published:** 2015-06-05

**Authors:** Safae Lamquami, Sanae Errarhay, Nisrine Mamouni, Chahrazad Bouchikhi, Abdelaziz Banani

**Affiliations:** 1 Obstetrics Gynecology Department I University, Hospital Hassan II, Fez, Morocco

**Keywords:** Dermatomyositis, paraneoplastic syndromes, breast cancer

## Abstract

Dermatomyositis (DM) is a rare connective corresponding to an inflammatory disease of skeletal muscles. Paraneoplastic origin must always be sought, primarily gynecological tumor in women, but the investigations are often made difficult by the fact that a primary tumor is often not detectable at the time of the cutaneous manifestations. This approach includes in addition to the monitoring report at regular intervals of 6 to 12 months for two years after diagnosis. We report a case of Dermatomyositis revealing breast cancer.

## Introduction

Paraneoplastic syndromes are a collection of disorders affecting an organ or tissue caused by cancer but occurring at a site distant from the primary or metastases. Dermatomyositis can occur in association with malignancy as a paraneoplastic phenomenon [1]. Dermatomyositis is a rare connective corresponding to an inflammatory disease of skeletal muscles. Paraneoplastic origin must always be sought, primarily gynecological tumor in women, but the investigations are often made difficult by the fact that a primary tumor is often not detectable at the time of the cutaneous manifestations.

## Patient and observation

**Figure 1:**
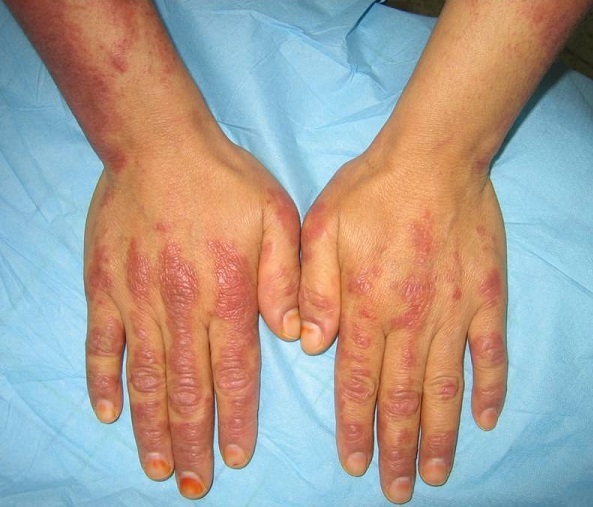
Gottron papules

**Figure 2:**
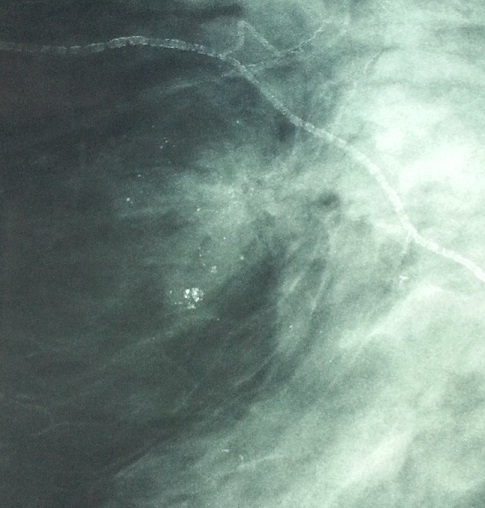
ACR5 lesion

38 year old woman with no significant medical history admitted to the dermatology department for management of dermatomyositis which restraint on clinical (Figure 1), biological, electromyographic and pathological criteria. An etiological looking for an initial paraneoplastic origin was back negative, outside a ACR3 lesion in the upper-inner quadrant of the left breast requiring radiological monitoring. The patient was put under high dose of steroids without a clear clinical improvement, mammography ultrasound control of six months revealed a ACR5 lesion in the same quadrant (Figure 2). Moreover, clinical breast screening examination remained normal. The echo guided biopsy objectified infiltrating ductal carcinoma grade II SBR. An additional surgical management was decided with achieving a conservative treatment after ultrasound localization of the lesion. The patient was put under adjuvant chemotherapy and radiotherapy with favorable Evolution with a 1 year follow-up.

## Discussion

Dermatomyositis (DM) is an uncommon idiopathic inflammatory myopathy that primarily affects skeletal muscle and skin with well characterized cutaneous findings. The estimated incidence of dermatomyositis is approximately 1/100,000. DM can affect both children and adults, and is more common in women than men (2:1). It has been well documented that DM carries an increased risk of malignancy and can present as a paraneoplastic syndrome to multiple types of underlying malignancies. DM is an autoimmune disorder characterized by inflammatory muscular and cutaneous disease. Clinically, it manifests with proximal muscle weakness and a typical skin rash. DM has been found to be a paraneoplastic phenomenon in 15% to 30% of adult patients [2]. In Malaysia, Tang and Thevarajah reported in 2010 that 47.4% of dermatomyositis patients had underlying malignancy [3].

The pathogenic relationship between dermatomyositis and cancer is not fully understood. It would appear that the regenerating cells that appear in muscles with myositis express high levels of the specific antigens of myositis, and that these are the same as those expressed in various cancers associated with inflammatory myopathies. The link between cancer and dermatomyositis would thus appear to be the expression of antigens common to the cancer and to muscle tissue in some patients with dermatomyositis [4]. Paraneoplastic dermatomyositis can precede, coincide with, or develop after the diagnosis of cancer. Of all the parameters studied, it would appear that those most consistently associated with a higher risk of cancer in patients with adult dermatomyositis are male gender and more advanced age. One clinical trait that has repeat-edly been related with paraneoplastic dermatomyositis in the literature is skin necrosis. In Europe, the cancers associated with adult dermatomyositis include, in order of frequency, ovary, lung, breast, colon and rectum, stomach, and pancreas. Other associated cancers include prostate and non-Hodgkin lymphoma.

The most common cancers associated with adult dermatomyositis in women are breast and ovary [4]. The risk of developing a specific type of cancer associated with dermatomyositis is unequal in different populations. Hill et al. reported on specific cancer types in dermatomyositis in Sweden, Denmark and Finland, where ovarian, lung and pancreatic cancers were reported as the three main types of cancers associated with dermatomyositis. In Scotland, lung cancer was the most common cancer related to dermatomyositis, while in Tunisia it was breast cancer [5]. Regarding the management of paraneoplastic dermato-myositis, our aim must always be to control the underlying neoplasm [4]. Topical emollients and steroids are important for all patients, and may control the skin lesions symptomatically until the tumor itself is treated and the DM regresses. If the tumor cannot be treated quickly and radically, then the patient will probably require oral corticosteroids. High-dose intravenous immunoglobulin has shown to be beneficial for recalcitrant DM. Hydroxychloroquine is quite effective in about 80% of DM patients when used as a steroid-sparing agent. Immunosuppressors, such as methotrexate, azathioprine or cyclosporin, may be effective in inducing or maintaining remission [6].

## Conclusion

The diagnosis of dermatomyositis requires etiological looking for a paraneoplastic origin. This approach includes in addition to the monitoring report at regular intervals of 6 to 12 months for two years after diagnosis.
